# Altered glycosylation associated with dedifferentiation of hepatocellular carcinoma: a lectin microarray-based study

**DOI:** 10.1186/s12885-020-6699-5

**Published:** 2020-03-06

**Authors:** Hiroomi Takayama, Masayuki Ohta, Yukio Iwashita, Hiroki Uchida, Yuki Shitomi, Kazuhiro Yada, Masafumi Inomata

**Affiliations:** 1 Department of Gastroenterological and Pediatric Surgery, Oita University Faculty of Medicine, 1-1 Idaigaoka, Hasama-machi, Yufu, Oita 879-5593 Japan; 2 Global Oita Medical Advanced Research Center for Health, Oita University, Oita, Japan

**Keywords:** Hepatocellular carcinoma, Dedifferentiation, High-mannose glycan, Lectin microarray, Mannosyl(α-1,3-)-glycoprotein β-1,2-*N*-acetylglucosaminyltransferas

## Abstract

**Background:**

Altered glycosylation associated with hepatocellular carcinoma (HCC) is well documented. However, few reports have investigated the association between dedifferentiation and glycosylation. Therefore, the aim of this study was to analyze glycosylation associated with dedifferentiation of HCC within the same nodule and to investigate glycosyltransferase related to the glycosylation.

**Methods:**

We analyzed resected HCC specimens (*n* = 50) using lectin microarray to comprehensively and sensitively analyze glycan profiles, and identify changes to glycosylation between well- and moderately-differentiated components within the same nodule. Moreover, we performed immunohistochemical staining of mannosyl(α-1,3-)-glycoprotein β-1,2-*N*-acetylglucosaminyltransferase (MGAT1), which is an essential glycosyltransferase that converts high-mannose glycans to complex- or hybrid-type *N*-glycans.

**Results:**

Four lectins from *Narcissus pseudonarcissus* agglutinin (NPA), Concanavalin A, *Galanthus nivalis* agglutinin, and *Calystegia sepium* agglutinin were significantly elevated in moderately-differentiated components of HCC compared with well-differentiated components, and all lectins showed binding specificity to high-mannose glycans. Therefore, these structures were represented to a greater extent in moderately-differentiated components than in well-differentiated ones. Immunohistochemical staining revealed significantly increased NPA expression and decreased MGAT1 expression in moderately-differentiated components. Low MGAT1 expression in moderately-differentiated components of tumors was associated with intrahepatic metastasis and had tendency for poor prognosis.

**Conclusion:**

Dedifferentiation of well-differentiated HCC is associated with an increase in high-mannose glycans. MGAT1 may play a role in the dedifferentiation of HCC.

## Background

Hepatocellular carcinoma (HCC) is a common cancer with poor prognosis [1, 2]. Liver cancer is the sixth most common type of cancer worldwide, and the fourth most common cause of cancer death [3]. HCC accounts for the most primary liver cancer. Therefore, exploring the mechanism of tumor progression and improving treatments for HCC are urgent requirements.

Glycosylation is involved in many essential biological processes such as cell differentiation, proliferation, and adhesion; immune response; and receptor activation. However, aberrant glycosylation results in many dysfunctions and diseases [4, 5]. For example, in many kinds of cancer, aberrant glycosylation such as fucosylation and sialylation, as well as altered expression of glycosyltransferase, which modulates glycosylation, have been reported [6–11]. In HCC, altered glycosylation, such as that of alpha fetoprotein (AFP)-L3—a core fucosylated AFP enhanced by fucosyltransferase—is well known [12, 13]. Moreover, fucosylated haptoglobin and fucosylated kininogen were also reported to be candidates for biological markers of HCC [14, 15].

Lectin microarray is a method capable of analyzing glycan profiles comprehensively and sensitively with 45 lectins utilizing lectin specificity to detect specific structures of glycans [16, 17]. Using this method, we reported the association between *Agaricus bisporus* agglutinin and colon cancer recurrence as well as between *Bauhinia purpurea* lectin and gastric cancer recurrence [18, 19]. In addition, we also reported that fucosylation was associated with the malignant transformation of intraductal papillary mucinous neoplasm of the pancreas [20].

HCC often comprises differentiated components—the so-called “nodule-in-nodule” appearance—which suggests multistep development [21, 22]. There have been several reports of dedifferentiation in HCC and altered gene expression of CAP, HSP70, p53, and β-catenin [23–25]. However, there are few reports of altered glycosylation associated with dedifferentiation. Therefore, in this study, we investigated glycosylation associated with the dedifferentiation of HCC within the same nodule, and glycosyltransferase related to the glycosylation.

## Methods

### Patients and tissue samples

We collected the clinical records and surgical specimens who underwent curative resection for HCC at the Department of Gastroenterological and Pediatric Surgery, Oita University Faculty of Medicine, from January 2006 to December 2015. Patients who underwent preoperative treatments such as transarterial chemoembolization and radiofrequency ablation were excluded from the study. In addition, the tumor size was limited to 3–10 cm to analyze well- and moderately-differentiated components within the same nodule. Well- and moderately-differentiated components in the same nodule were histologically distinguished by two pathologists on the basis of typical characteristics using hematoxylin and eosin (HE) stain according to the General Rules for the Clinical and Pathological Study of Primary Liver Cancer [26]. Finally, 50 patients were enrolled in the study. We also collected pathological data including number of tumors, tumor size, intrahepatic metastasis, portal vein invasion, venous invasion, arterial invasion, biliary invasion, capsule invasion, and serosal invasion. All clinical data and tissue samples were collected after obtaining informed consent from the included patients.

### Sample preparation and lectin microarray

Fifty tissue samples were prepared for laser microdissection by fixing in formalin, embedding in paraffin, then sectioning at a thickness of 5 μm. The sections were placed on dedicated glass slides and stained with HE after deparaffinizing. Well- and moderately-differentiated components were extracted from the same nodule using laser microdissection. Each section was 5 × 10^6^ μm^2^ to equalize the tissue volume. Lectin microarray was performed as previously described [18, 20]. In brief, sections were sonicated with Bioruptor UCW-310 (Cosmobio, Co., Ltd., Tokyo, Japan). Proteins were extracted with Zeba Desalt Spin Columns (Thermo Scientific Ltd., Rockford, IL, USA), labeled with cyanine 3 fluorescent dye, and transferred onto Lectip (GlycoTechnica Ltd., Yokohama, Japan) with seven wells containing 45 lectins. The list of lectins and their specificities to glycans is available from the manufacturer [27]. Fluorescent images were obtained with the Glycostation Reader 1200 (GlycoTechnica Ltd.) using the evanescent-wave excitation method [28]. Data were analyzed using Glycostation Tool Pro Suite 1.5 (GlycoTechnica Ltd.). Signal intensities were measured in triplicate and normalized across the 45 lectins by setting the average intensity of the 45 lectins to 100.

### Lectin staining and immunohistochemistry

Fifty formalin-fixed and paraffin-embedded tissues were sectioned at a thickness of 3 μm for lectin staining and immunohistochemistry, as described previously [18, 20]. For lectin staining, the sections were incubated with biotinylated *Narcissus pseudonarcissus* agglutinin (NPA) (BA-8006-1, EY Laboratories, Inc., San Mateo, CA, USA) and then processed using the VECTASTAIN Elite ABC kit (Vector Laboratories, Inc., Burlingame, CA, USA) according to the manufacturer’s instructions. For immunohistochemical analysis, mannosyl(α-1,3-)-glycoprotein β-1,2-*N*-acetylglucosaminyltransferase (MGAT1) (15103–1-AP, Proteintech, Inc., Chicago, IL, USA)—an essential glycosyltransferase that converts high-mannose type *N*-glycans to complex- or hybrid-type *N*-glycans—was used as the primary antibody. Staining intensity was scored in duplicate by two pathologists as follows: negative, 0 point; weak (< 10% positive staining), 1 point; moderate (10–50% positive staining), 2 points; and strong (> 50% positive staining), 3 points [29, 30]. The clinicopathological outcomes of the patients were blinded to the pathologists. In case of discrepancy in provisional scores between the pathologists, the final scores were determined through their consensus. On the basis of scores, tumors were divided into two groups with low (score 0 or 1) or high (score 2 or 3) MGAT1 expression in the moderately-differentiated components. Overall survival (OS) and disease-free survival (DFS) were estimated, and patients were also divided into within (*n* = 27) and beyond (*n* = 23) the Milan criteria groups for analysis [31].

### Statistical analysis

All statistical analyses were performed using SPSS, version 20 statistical software (SPSS Inc., Chicago, IL, USA). Data were expressed as the mean ± standard error of the mean (SEM). Differences between well- and moderately-differentiated components in lectin microarray signal and staining intensities were analyzed by Wilcoxon signed rank test. Other categorical variables were analyzed using Fisher’s probability test, and continuous variables using Mann–Whitney U test. OS and DFS were analyzed using the Kaplan–Meier method, and compared using the log-rank test. The level of probability was set at *P* < 0.05 as statistically significant.

## Results

A total of 45 lectin signal patterns were analyzed comprehensively between well- and moderately-differentiated components of HCC. Among them, four lectins of NPA, Concanavalin A (ConA), *Galanthus nivalis* agglutinin (GNA), and *Calystegia sepium* agglutinin (Calsepa) were significantly increased in moderately-differentiated components compared with well-differentiated components (Table 1). All the lectins showed specificity to high-mannose glycan structures and none were significantly decreased by dedifferentiation.

**Table 1:** Differences in lectin microarray signal intensity between well- and moderately-differentiated components of HCC (*n* = 50)

Lectin	Well-differentiated			Moderately-differentiated			***P***-value
LTL	14.6	±	0.8	13.9	±	1.1	0.518
**PSA**	48.4	±	2.6	52.5	±	3.9	0.406
**LCA**	73.8	±	3.3	78.6	±	4.1	0.622
**UEA-I**	10.0	±	1.5	8.1	±	1.3	0.285
**AOL**	81.7	±	7.2	72.7	±	6.5	0.157
**AAL**	101.9	±	6.7	89.9	±	6.8	0.076
**MAL-I**	11.4	±	0.9	9.6	±	1.1	0.095
**SNA**	232.6	±	9.0	223.2	±	9.9	0.431
**SSA**	233.6	±	11.4	222.1	±	11.5	0.215
**TJA-I**	319.2	±	13.4	303.7	±	16.7	0.224
**PHA(L)**	4.3	±	0.7	4.7	±	0.7	0.461
**ECA**	6.6	±	1.0	5.5	±	0.7	0.572
**RCA120**	112.7	±	8.6	117.4	±	16.7	0.267
**PHA(E)**	82.9	±	5.9	77.9	±	5.4	0.958
**DSA**	323.0	±	10.6	304.7	±	7.9	0.112
**GSL-II**	5.7	±	1.4	7.4	±	2.3	0.737
**NPA**	137.7	±	11.3	149.8	±	12.6	0.049^*^
**ConA**	212.6	±	15.4	243.2	±	19.2	0.008^*^
**GNA**	63.2	±	4.0	73.1	±	4.6	0.028 ^*^
**HHL**	23.3	±	1.7	25.6	±	2.4	0.824
**ACG**	126.3	±	9.6	115.8	±	8.3	0.275
**TxLC-I**	48.1	±	5.1	44.9	±	4.4	0.553
**BPL**	11.4	±	1.4	13.8	±	1.7	0.338
**TJA-II**	45.7	±	5.1	43.9	±	3.8	0.735
**EEL**	3.4	±	0.6	3.3	±	0.6	0.781
**ABA**	93.4	±	5.3	96.3	±	8.0	0.595
**LEL**	393.0	±	7.6	393.0	±	8.4	0.757
**STL**	490.4	±	13.3	498.9	±	11.9	0.472
**UDA**	359.6	±	7.5	352.0	±	8.0	0.434
**PWM**	7.8	±	0.8	8.2	±	1.0	0.648
**Jacalin**	132.5	±	4.5	137.4	±	5.6	0.443
**PNA**	3.1	±	0.5	2.9	±	0.5	0.825
**WFA**	10.7	±	1.3	10.5	±	1.1	0.992
**ACA**	56.7	±	2.2	61.2	±	2.8	0.118
**MPA**	32.3	±	2.5	30.8	±	2.3	0.636
**HPA**	25.3	±	1.9	26.3	±	2.3	0.731
**VVA**	6.2	±	0.9	6.2	±	0.9	0.831
**DBA**	6.9	±	0.9	7.1	±	1.5	0.314
**SBA**	6.2	±	1.0	6.3	±	0.9	0.688
**Calsepa**	342.9	±	23.7	363.5	±	23.1	0.039^*^
**PTL-I**	4.2	±	0.8	4.9	±	0.7	0.170
**MAH**	19.5	±	1.0	16.9	±	1.0	0.073
**WGA**	149.8	±	5.8	147.1	±	5.3	0.612
**GSL-I-A4**	8.9	±	1.1	10.1	±	1.3	0.659
**GSL-I-B4**	8.3	±	0.9	8.0	±	1.0	0.800

Mean ± SEM, ^*^P < 0.05 (statistically significant)

**Fig. 1:**
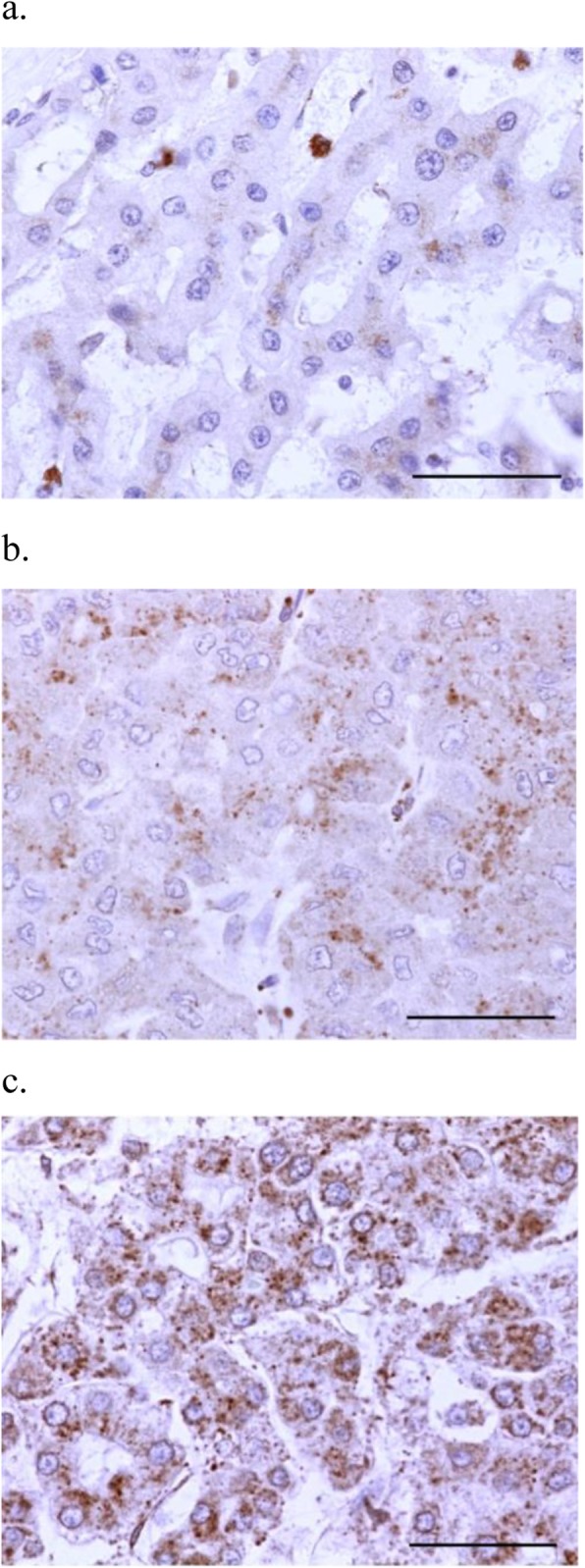
Representative lectin staining of NPA in HCC specimens (× 400). Intensity: (**a**) weak (1 point), (**b**) moderate (2 points), and (**c**) strong (3 points). No specimen showed negative staining (0 point). Scale bar indicates 50 μm

**Fig. 2:**
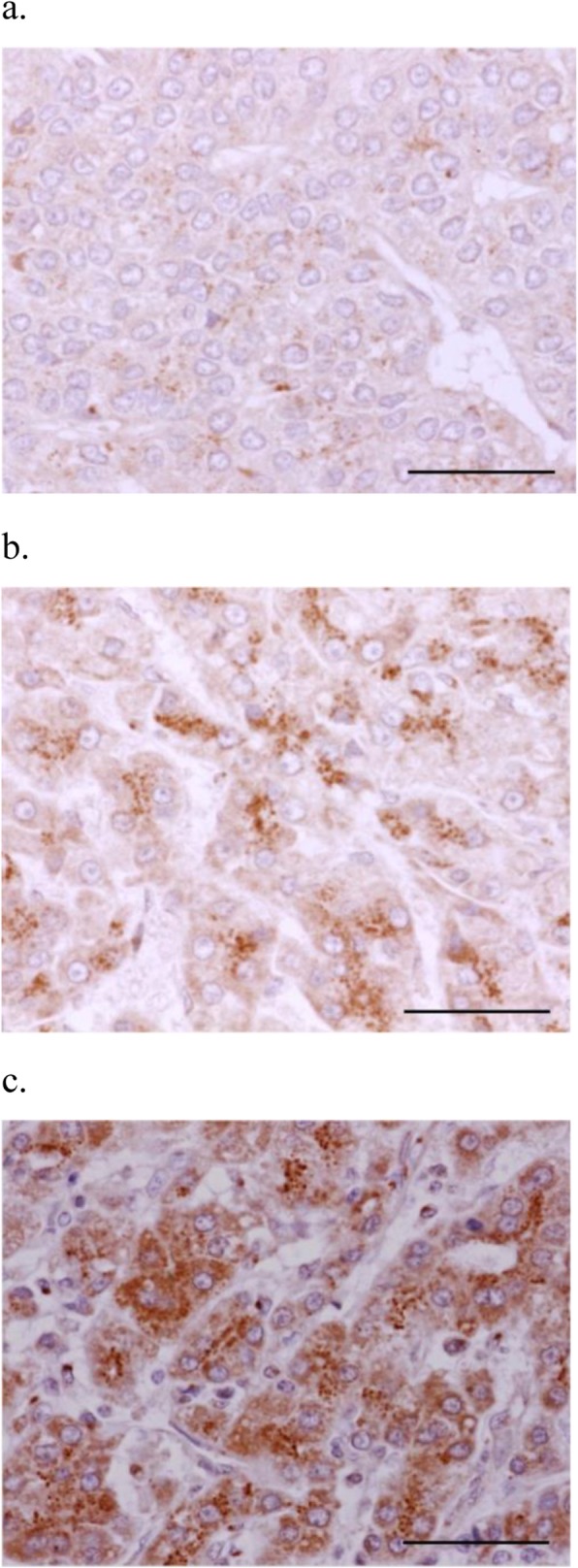
Representative immunohistochemical staining of MGAT1 in HCC specimens (× 400). Intensity: (**a**) weak (1 point), (**b**) moderate (2 points), and (**c**) strong (3 points). No specimen showed negative staining (0 point). Scale bar indicates 50 μm

**Fig. 3:**
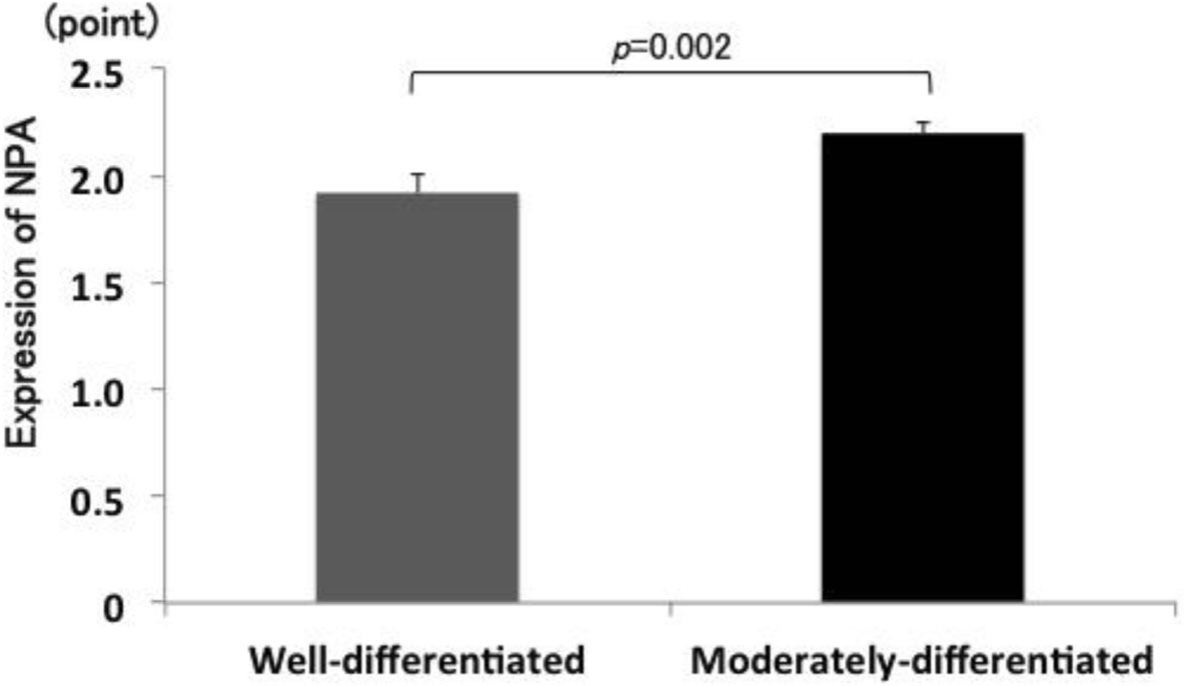
Differences in NPA staining scores between well- and moderately-differentiated components of HCC

**Fig. 4:**
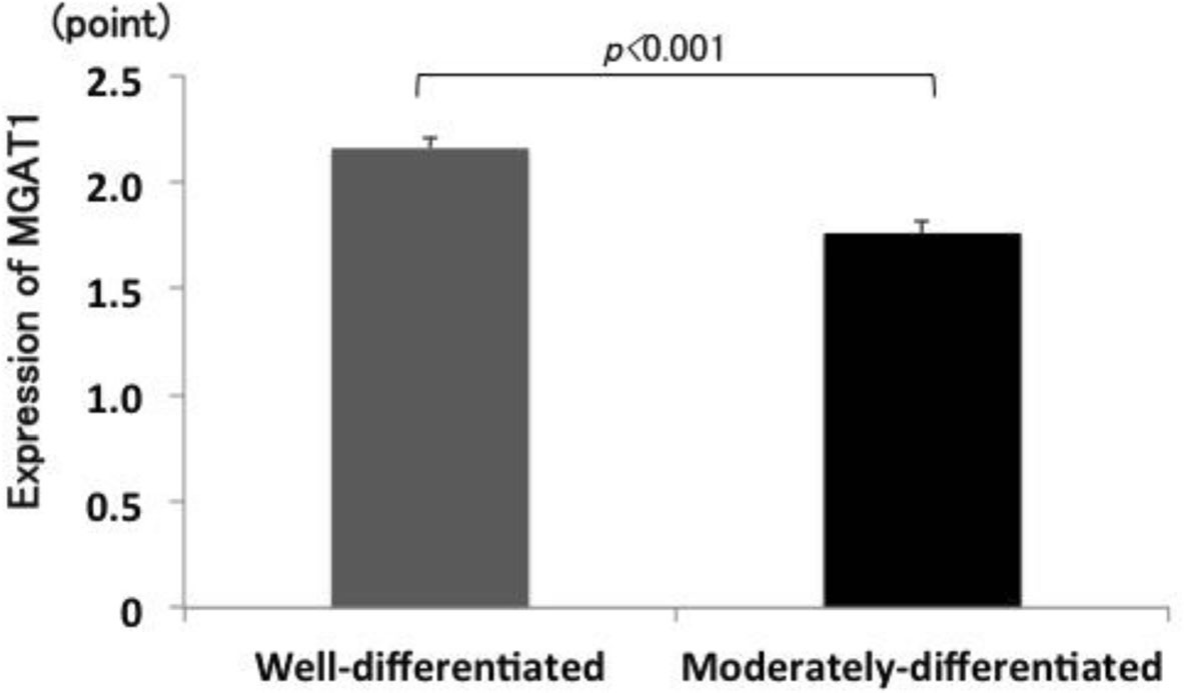
Differences in MGAT1 staining scores between well- and moderately-differentiated components of HCC

Representative staining of NPA and MGAT1 is presented in Figs. 1 and 2. NPA staining scores were significantly increased in moderately-differentiated components compared with those in well-differentiated components (*p* = 0.002) (Fig. 3). In contrast, MGAT1 staining scores were significantly decreased in moderately-differentiated components compared with those in well-differentiated components (*p* < 0.001) (Fig. 4).

**Fig. 5:**
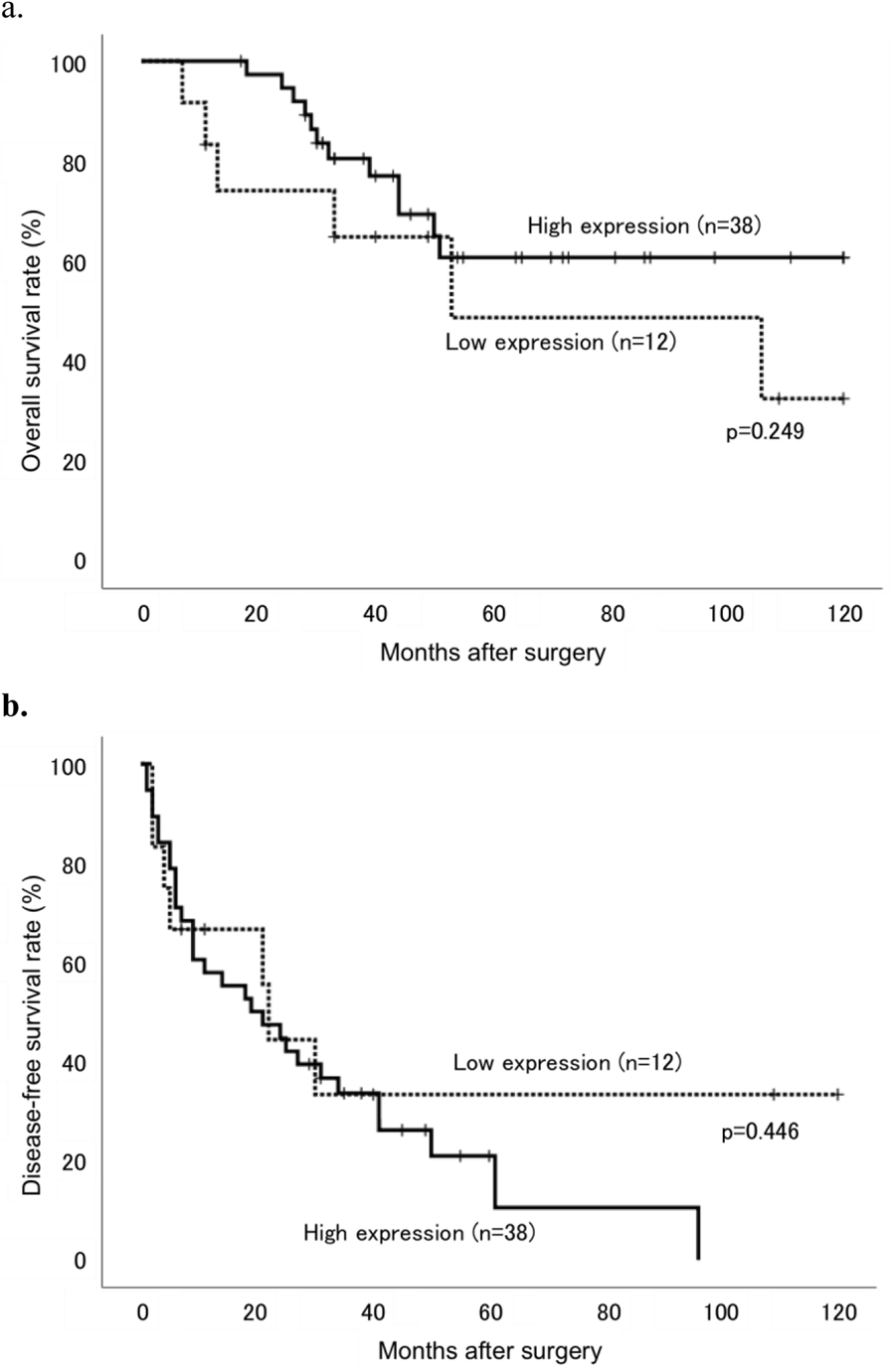
Kaplan–Meier curves of (**a**) overall survival (OS) and (**b**) disease-free survival (DFS) rates in patients with HCC after surgery stratified according to MGAT1 expression levels in the moderately-differentiated components. Patients with low expression tumors are represented by dotted lines and those with high expression tumors are represented with solid lines

**Fig. 6:**
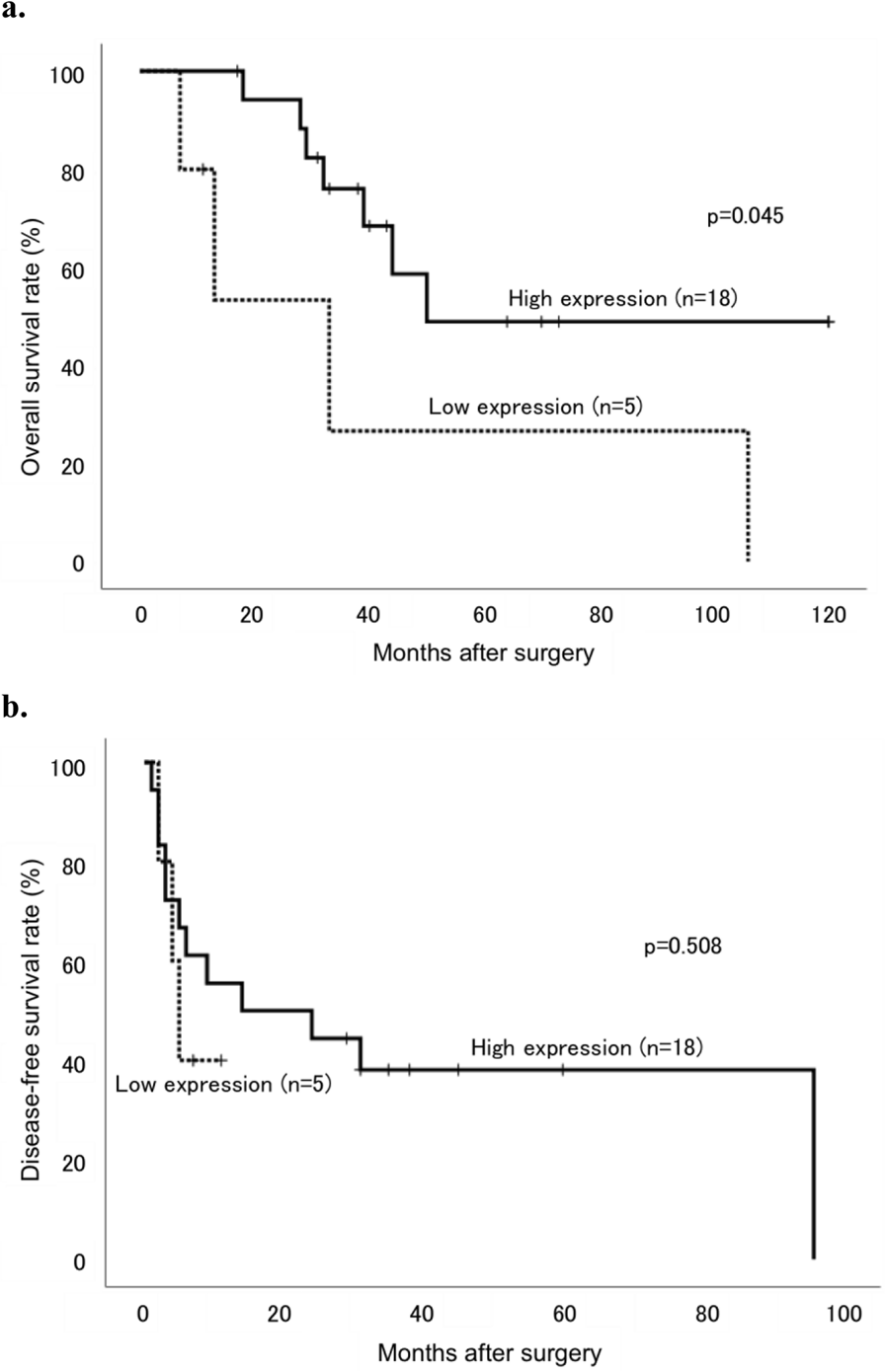
Kaplan–Meier curves of (**a**) OS and (**b**) DFS rates in HCC patients beyond the Milan criteria stratified according to MGAT1 expression levels in the moderately-differentiated components. Patients with low expression tumors are represented by dotted lines and those with high expression tumors are represented with solid lines

Low and high MGAT1 expression in the moderately differentiated components was noted in 12 and 38 patients, respectively. Table 2 presents the associations between the clinicopathological findings and MGAT1 expression levels. Intrahepatic metastasis alone was significantly correlated with low MGAT1 expression group (*p* = 0.031). OS analysis revealed no significant differences between the low and high MGAT1 expression groups (*p* = 0.249); however, the prognosis tended to be poor in the low MGAT1 expression group (Fig. 5a). DFS showed neither significant differences nor any trend (*p* = 0.446, Fig. 5b). Similarly, patients in the within the Milan criteria group, there were no significant differences in OS (*p* = 0.796) and DFS (*p* = 0.145) between patients with low and high MGAT1 expression. Meanwhile, in patients in the beyond the Milan criteria group, those with low MGAT1 expression group showed significantly poorer prognosis in terms of OS than those with high MGAT1 expression group (*p* = 0.045, Fig. 6a). DFS showed no significant differences between the groups (*p* = 0.508, Fig. 6b).

**Table 2:** Association between the clinicopathological characteristics and MGAT1 expression in moderately-differentiated components of HCC

Clinicopathological characteristics		Total	MGAT1 expression		***P***-value
		n = 50	High (***n*** = 38)	Low (***n*** = 12)	
**Age (years)**			72.5 ± 1.3	71.3 ± 2.0	0.459
**Sex**	Male	38	31	7	0.100
	Female	12	7	5	
**Number of tumor**			1.7 ± 0.3	1.3 ± 0.3	0.176
**Tumor size (mm)**			45.3 ± 3.7	59.7 ± 7.9	0.086
**Intrahepatic metastasis**	–	40	33	7	0.031*
	+	10	5	5	
**Portal vein invasion**	–	43	34	9	0.208
	+	7	4	3	
**Venous invasion**	–	40	31	9	0.619
	+	10	7	3	
**Arterial invasion**	–	49	37	12	0.570
	+	1	1	0	
**Biliary invasion**	–	49	37	12	0.570
	+	1	1	0	
**Capsule invasion**	–	26	21	5	0.411
	+	24	17	7	
**Serosal invasion**	–	41	33	8	0.113
	+	9	5	4	
**Milan criteria**	Within	27	20	7	1.000
	Beyond	23	18	5	
**Recurrence**	–	26	8	5	0.256
	+	24	30	7	
**Death**	–	41	26	6	0.309
	+	9	12	6	

Mean ± SEM, *P < 0.05 (statistically significant)

## Discussion

The present study is the first to demonstrate altered glycosylation associated with dedifferentiation in HCC using lectin microarray. The signal intensities of four lectins, including NPA, ConA, GNA, and Calsepa, were significantly increased in moderately-differentiated components compared with those in well-differentiated components. Since lectins bind to glycan structures, these structures are in fact represented to a greater extent in cells. All four lectins showed binding specificity to high-mannose glycan structures, thereby the glycans were significantly increased in moderately-differentiated components compared with well-differentiated components in HCC. NPA—one of the elevated lectins that binds high-mannose glycans—has been reported to be increased in gastric cancer cell lines [32]. Therefore, we performed NPA staining to confirm elevated high-mannose glycan expression and demonstrated that increased high-mannose glycans expression was associated with decreased MGAT1 expression.

Among high-mannose structures, NPA binds to Manα1–6Man, ConA binds to Manα1–6(or Manα1–3)Man, GNA binds to Manα1–3Man, and Calsepa binds to Man2–6 and *N*-glycans including bisecting GlcNAc [16]. All examined lectins showed specificity to high-mannose glycan structures. High-mannose-type glycans, which are a type of *N*-glycan, are attached to proteins and play essential roles in the transfer of correctly folded proteins from the endoplasmic reticulum to the Golgi apparatus [33]. Several enzymes involved in *N*-glycan processing are candidates for the mechanism of increased high-mannose glycans. MGAT1 is a key glycosyltransferase that initiates the conversion of high-mannose-type glycans to complex- and hybrid-type *N*-glycans and is significantly associated with human homeostasis [34]. Recently, MGAT1 has also been proposed to play a substantial role in tumor immunity [35]. Lack of this enzyme results in an abundance of high-mannose glycans [36]. Decreased expression of the MGAT1 gene in breast cancer tissue was associated with poor prognosis [37]. Similarly, decreased MGAT1 expression was observed in HCCLM3 cells, which show a higher metastatic potential than Hep3B cells [38]. Also, decreased expression of mannosidase alpha class 1A member 1 (MAN1A1), which trims α-1,2-linked mannose residues from Man9 high-mannose glycan in the Golgi apparatus, could also result in an increase in high-mannose glycans, and mannosyltransferase may increase the levels of high-mannose glycans. However, few studies have reported associations between enzymes and cancer. In the present study, MGAT1 expression decreased with dedifferentiation of HCC, potentially resulting in an increase in the levels of high-mannose glycans. In addition, low MGAT1 expression in moderately-differentiated components of tumors was associated with intrahepatic metastasis and tendency of poor prognosis. In patients within the Milan criteria, there were no significant differences in OS and DFS between the low and high MGAT1 expression groups, but there were significant differences in OS in patients beyond the Milan criteria.

Many studies have reported increases in high-mannose glycans in cancer, including in HCC model rats [39]. In addition, an epithelial–mesenchymal transition (EMT)-induced HCC cell line, that indicates a metastatic potential, also showed an increase in high-mannose glycans compared with an HCC cell line without EMT induction [40]. Other studies have shown abundant expression of high-mannose glycans in colorectal cancer cell lines including moderately- and poorly-differentiated cell lines and metastatic cell lines, as well as in colorectal cancer tissues [41, 42]. Glycans were also increased in breast cancer tissues compared with normal tissues. Furthermore, stage II and III cancer tissues showed significantly higher glycan expression than stages 0 and I tissues [43]. In the present study, high-mannose glycans were increased according to HCC dedifferentiation; therefore, increased high-mannose glycan expression may be associated with high-grade HCC malignancy.

Our study has some limitations. First, the number of specimens studied was small. Second, we did not assess MGAT1 function in HCC dedifferentiation. Finally, we did not examine the expression of other enzymes involved in *N*-glycan processing. Therefore, further studies are necessary. If demonstrated to have an apparent function in HCC dedifferentiation, MGAT1 can serve as a potential target for HCC treatment in the future.

## Conclusions

In conclusion, dedifferentiation of well-differentiated HCC is associated with increased high-mannose glycans. Furthermore, MGAT1 may play a role in HCC dedifferentiation.

## Data Availability

The datasets used and/or analyzed during the current study are available from the corresponding author upon reasonable request.

## Abbreviations

AFP: Alpha fetoprotein
Calsepa: *Calystegia sepium* agglutinin
ConA: Concanavalin A
DFS: Disease-free survival
EMT: Epithelial–mesenchymal transition
GNA: *Galanthus nivalis* agglutinin
HCC: Hepatocellular carcinoma
HE: Hematoxylin and eosin
MGAT1: Mannosyl(α-1,3-)-glycoprotein β-1,2-*N*-acetylglucosaminyltransferase
NPA: *Narcissus pseudonarcissus* agglutinin
OS: Overall survival
SEM: Standard error of the mean
